# Clinical reasoning skill of nurses working in teaching medical centers in dealing with practical scenarios of King’s model concepts

**DOI:** 10.1186/s12909-024-05256-x

**Published:** 2024-03-13

**Authors:** Seyyed Reza Borzo, Fatemeh Cheraghi, Mahnaz Khatibian, Marzieh Jahani Sayad Noveiri

**Affiliations:** 1 Department of Medical Surgical Nursing, Chronic Disease (Home Care) Research Center, School of Nursing and Midwifery, Hamadan University of Medical Science, Hamadan, Iran; 2 Department of Pediatric Nursing, Chronic Disease (Home Care) Research Center, School of Nursing and Midwifery, Hamadan University of Medical Science, Hamadan, Iran; 3 Department of Medical Surgical Nursing, Maternal and Child Care Research Center, School of Nursing and Midwifery, Hamadan University of Medical Science, Hamadan, Iran; 4 Department of Medical Surgery, School of Nursing and Midwifery, Guilan University of Medical Sciences, Rasht, Iran

**Keywords:** Nurses, Clinical reasoning, King’s model

## Abstract

**Background:**

The present study was conducted to determine clinical reasoning of nurses working in teaching medical centers in dealing with practical scenarios of King’s concepts.

**Methods:**

The study population in this cross-sectional descriptive-analytical study comprised 133 nurses. Data were collected using scenarios based on the King’s model. Data were analyzed in SPSS-16.

**Findings:**

Mean age of the participating nurses was 27.71 ± 8.1 years.The clinical reasoning score was less than average in most participating nurses, and had a significant relationship with education(*P* < 0.05), service ward(*P* < 0.001)and organizational position(*P* < 0.05). In the multivariate analysis of factors relating to clinical reasoning, higher education level (B = 9.5, *P* = 0.018) and organizational position (B = 4.3, *P* = 0.017) were predictors of clinical reasoning score.

**Discussion:**

Existing nursing models such as King’s, which is closely related to clinical reasoning, can be used more in educational and clinical systems, and as a clinical guide for promoting the clinical reasoning of nurses and students.

## Introduction

As the largest group of healthcare providers at the pinnacle of the healthcare delivery pyramid [1, 2] nurses frequently encounter situations in which they need to understand the position of patients, their accompaniment and themselves as healthcare providers. The dynamic, and variable conditions of nurses’ workplace plus patients’ uncertain and changing situations require nurses to be competent decision-makers who can combine their technical skills and professional knowledge to make accurate and correct clinical judgments about patients’ health status and identify and solve patients’ nursing-related problems within a multidisciplinary team [3]. Clinical decision-making skills can affect the quality of care more than any other factor [4]. Clinical reasoning is among the most essential features of care that focus on simulating and analyzing clinical situations to facilitate better decisions for and management of patients [5]. Nurses’ reasoning in different situations and the decisions made and practiced based on such logic directly affect the process of treatment and recovery, facilitate the process of treatment, increase patient satisfaction, and ultimately affect the treatment economy positively [6].

Generally, clinical reasoning is a logical process of collecting key points of information, understanding a patient’s problems and condition, planning and implementing interventions, assessing interventions, and providing feedback throughout the learning process [5]. Clinical reasoning in nursing is defined as a cognitive process and strategies used to understand information, identify patients’ problems, and a way of clinical thinking about clinical skills [7]. Everyone knows that nurses make important clinical decisions with significant effects on patient care and their professional practice [4]. Clinical reasoning is based on recognizing the right key points, which can be found in physiological and psychological changes in patients, or through physical examination, and patient history, and are understandable according to the body of knowledge and philosophical beliefs. Obtaining key points is affected by experience, application of knowledge in decision-making, stress, self-esteem, etc. In the absence of correct key points, incorrect reactions occur. Nurses with weak clinical reasoning cannot successfully understand the patients, identify their condition, make the right decisions about them, and ultimately save them [8, 9].

Teaching care and health for assessing skills needed for a professional role such as clinical reasoning and decision-making skills requires a particular test for assessing clinical capabilities [10]. The test for clinical reasoning is different from that for skills and knowledge, as it aims to assess the individual’s problem-solving ability; in fact, clinical reasoning and its test focus on action and decision-making in clinical situations [11]. Therefore, placing the examinee in a particular scenario appears to be an appropriate way to obtain information. To have the right understanding of nurses’ clinical reasoning, nursing frameworks and models can used as a scientific basis and an appropriate method [12, 13].

Every nursing model and theory has its perspective on nurses and the nursing profession and tries to improve nursing and community health with its unique perspective. Using a specific nursing model unifies nurses’ understanding and practice in different fields related to human and their health-related needs, and entails more coherent and better nursing care [14, 15]. The perspective of any clinical theories can be used to address nursing. One of the models in this field is Imogene King’s model. This conceptual framework, referred to as “The general framework of systems and the theory of goal attainment”, has had extensive applications in different fields of nursing (clinical, educational, research, and management) [14, 16]. King’s theory has been used in decision-making studies, the promotion of self-care behaviors, and communication between nurses and patients, as well as patient fall prevention programs and other cases [17, 18]. Adlib has investigated the application of this theory in relationships with patients [19]. King’s theory provides a practical guide for clinical nurses, especially for communicating with, identifying, and understanding patients, patient-nurse actions and reactions, and ultimately the patient-nurse interactions, it appears that the concepts of this theory and understanding nurse’s perception of these concepts effectively help nurses with clinical reasoning about patients and their condition. King’s conceptual system covers the Meta paradigm concepts of human, environment, health, and nursing, and each concept is classified according to its Meta paradigm symbol. The Meta paradigm concept of humans in King’s conceptual system is expressed in terms of personal, interpersonal, and social systems, each of which is multidimensional. The concept of a **personal** system has seven dimensions, including perception, self, growth and development, body image, time, space and learning. The concept of interpersonal system embodies five dimensions, including, interaction, communication, transaction, role, stress, and coping, and the concept of **social** system has five dimensions, including organization, authority, power, status, decision-making, and control [20, 21]. Given the stages of nursing care, from patient examination to practicing procedures, nurses need to consider each concept and ultimately establish a mutual interaction between the patient and themselves [19]. King’s goal attainment theory defines a process of interaction in which patients and nurses set goals together and agree and interact on how to achieve those goals [17]. This theory focuses on respect for patients and the patient-nurse relationship, which emphasizes information exchange, goal setting, and patient treatment. Therefore, there needs to be a positive correlation between trust and patient satisfaction [22]. Such interactions allow patients to take responsibility for and actively participate in the proposed treatment for positive change. It also gives nurses the perspective to value patients’ opinions and requests in this framework and to cooperate with patients to achieve a common goal for treatment and to create appropriate interaction. Nurses have clinical reasoning and provide patient care in this framework. Therefore, the theory of achieving the goal is an important strategy in nursing patients [23], in the present study, this theory is used to examine nurses’ clinical reasoning skills.

Therefore, attending to the concepts of King’s theory will help provide a better patient care program. Placing nurses in situations and scenarios they face in the workplace every day can show how nurses pay attention and focus on these concepts while working, and reveals how service-providing nurses deal with these matters in their environment and practice, and how service recipients can be sure of receiving these services. The aim of this study is to the reasoning ability of nurses based on King’s practical concepts. It is hoped that a small step will be taken in the direction of benefiting from common nursing theories and the development of clinical nursing knowledge.

## Method

### Sample and design

The present cross-sectional descriptive-analytical study was conducted in one of the teaching medical centers in Guilan Province, which has all general and specialized departments in 2019. The statistical population included all nurses working in this teaching medical center, 140 nurses were included in the study by census method. The data was collected using a researcher-made questionnaire based on King’s theory. Data were collected after obtaining approval of the ethics committee.

### Procedure

This study was conducted after receiving the code of ethics in a teaching medical center in Iran. In this study, all nurses working in this medical center were included in the study. The right to withdraw from the study and the confidentiality of the data was given to the nurses, and after obtaining informed consent, a two-part researcher-made questionnaire was given to the samples.

### Measures

The questionnaire was in two parts. In the first part, participants’ demographic data including gender, age, education, organizational position, and service ward were collected, and the second part consisted of 17 scenarios, each about one of the concepts of King’s theory (perception, self, growth and development, body image, time, space and learning, interaction, communication, transaction, role, stress and coping, organization, authority, power, status, decision-making and control). A score of -2, -1, + 1, and + 2 can be given to each scenario depending on the level of attention/or lack of attention to the concepts. Participants were asked to carefully review each scenario and choose one option only, and if none of the options matched their intended answer, choose the nearest option to their response; not to leave any scenario unanswered, not to choose more than one option, not to get help from a colleague or consult with others. The scenarios of this study were provided to professors and nurses by the research team using King’s texts and theory, and the necessary corrections were made. Also, for the validity of the qualitative content, the opinions of 5 professors who had experience in this field were used. Cronbach’s alpha was used to check the reliability, and Cronbach’s alpha score for the entire questionnaire was 0.780. The study inclusion criteria included being a clinical nurse, head nurse, or supervisor. The research samples that did not answer all the questions were excluded from the study due to not achieving the research objectives.

### Ethical considerations

The principles of the revised Declaration of Helsinki, a statement of ethical principles that direct physicians and other participants in medical research involving human subjects, were considered in all parts of the present study. All participants signed the informed consent to participate in the study. The participants were assured that all their personal information would remain confidential and that they were free to withdraw at any study stage. We provided them with sufficient information as to the anonymity and confidentiality of their information. Moreover, the Research Ethics Committee of Hamadan University of Medical Sciences, Hamadan, Iran approved the study with the code of IR.UMSHA.REC.1396.777.

### Statistical analyses

The data collected were analyzed in SPSS-16 using descriptive statistics of frequency distribution, mean, standard deviation, and inferential statistics of independent t and Chi-squared tests. One-way ANOVA and Tukey’s tests were used to compare factors associated with the score of nurses’ clinical reasoning.

## Results

Of the sample size of 140 nurses, 133 completed the questionnaires and entered the study. The Kolmogorov-Smirnov test (0.63) confirmed the normal distribution of data. Participants’ mean age was 27.71 ± 8.1 years, the majority were female (98.5%) with bachelor’s degree education (97%). None of the participants had any training about King’s theory and its systems. Generally, the clinical reasoning score had a significant relationship with education (*P* < 0.05), service ward (*P* < 0.001), and organizational position (*P* < 0.05), such that those with master’s degree had higher mean clinical reasoning scores compared to those with bachelor’s degree, and supervisors and head-nurses also had higher mean scores than nurses. In terms of service ward, the highest mean clinical reasoning score belonged to the pediatric ward (37.6) followed by the nursing office (36.7), while gynecology ward had the lowest mean score (21.1). Table 1 shows the descriptive statistics.

**Table 1:** The demographic details of the participants in the study of “Clinical reasoning skill of nurses working in teaching medical centers in dealing with practical scenarios of King’s concepts”

		Total_score						P
		Count	Column N %	Mean	Standard Deviation	Minimum	Maximum	
Age	20–25	34	25.6%	26.38	7.02	12.00	38.00	0.243
	26–30	23	17.3%	25.78	8.63	10.00	42.00	
	31–35	32	24.1%	28.34	8.40	12.00	42.00	
	> 35	44	33.1%	29.30	8.01	14.00	43.00	
	Total	133	100.0%	27.71	8.01	10.00	43.00	
Education	Bachelor	129	97.0%	27.37	7.88	10.00	43.00	0.005
	Master	4	3.0%	38.75	2.63	36.00	41.00	
	Total	133	100.0%	27.71	8.01	10.00	43.00	
Work experience	0–5	44	33.1%	26.84	7.85	10.00	42.00	0.416
	6–10	38	28.6%	26.95	8.16	12.00	43.00	
	11–15	33	24.8%	28.39	8.46	14.00	41.00	
	> 15	18	13.5%	30.22	7.22	18.00	43.00	
	Total	133	100.0%	27.71	8.01	10.00	43.00	
Gender	male	2	1.5%	32.00	4.24	29.00	35.00	0.448
	female	131	98.5%	27.65	8.05	10.00	43.00	
	Total	133	100.0%	27.71	8.01	10.00	43.00	
Department	General	14	10.5%	29.36	4.68	20.00	34.00	< 0.001
	Supervisor unit	4	3.0%	36.75	0.96	36.00	38.00	
	pediatric	11	8.3%	37.64	3.70	32.00	43.00	
	Emergency	30	22.6%	25.33	6.95	14.00	39.00	
	Dialysis	8	6.0%	22.25	6.27	14.00	30.00	
	Operation room	11	8.3%	25.73	8.20	14.00	37.00	
	Maternity ward	11	8.3%	23.91	4.44	19.00	35.00	
	Men’s surgery	11	8.3%	26.36	5.94	18.00	36.00	
	Gynecological surgery	15	11.3%	21.13	9.05	10.00	42.00	
	CCU	9	6.8%	33.44	5.15	28.00	43.00	
	ICU	9	6.8%	35.78	4.84	27.00	41.00	
	Total	133	100.0%	27.71	8.01	10.00	43.00	
Post	Nurse	128	96.2%	27.33	7.91	10.00	43.00	0.005
	Head nurse/ Supervisor	5	3.8%	37.60	2.07	36.00	41.00	
	Total	133	100.0%	27.71	8.01	10.00	43.00	

## Main analysis

The clinical reasoning ability of most nurses was about 25% (48.12%), and none of the nurses scored 100% in all concepts of clinical reasoning. Meanwhile, 1.5% of nurses scored zero in dealing with scenarios (Table 2).

**Table 2:** Clinical reasoning power percentage based on King Model

Clinical reasoning power		Mean	Standard Deviation	95.0% Lower CL	95.0% Upper CL
Total_score		27.71 ± 8.01(10.0–43)		26.34	29.09
Total_status	clinical reasoning power 0	7.00(5.3)	5.30	2.40	10.10
	clinical reasoning power 25%	64.00(48.1)	48.10	39.70	56.60
	clinical reasoning power 50%	60.00(45.1)	45.10	36.80	53.60
	clinical reasoning power 75%	2.00(1.5)	1.50	0.30	4.70
	clinical reasoning power 100%	0.00(00)	0.00	-	-
	Total	133.00	100.00	-	-

**Diagram 1.**
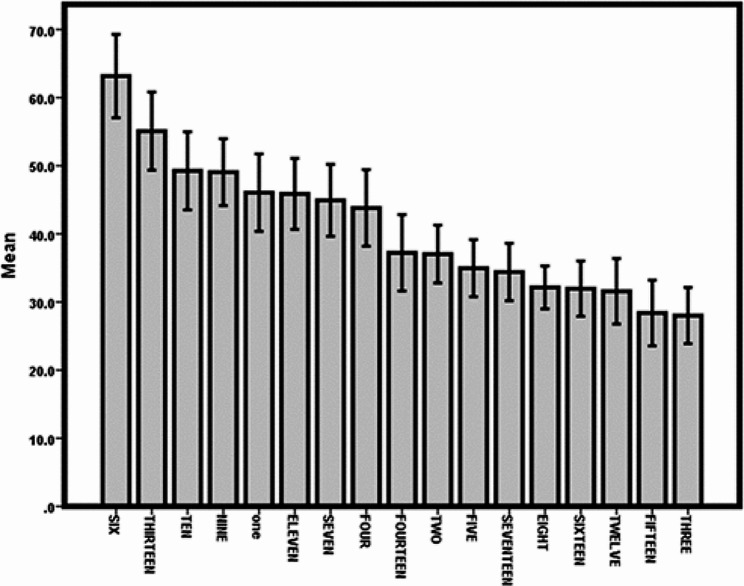
Average score in each scenario

According to the results, scenario 6 (relating to space) scored the highest, and scenario 15 (relating to patient authority) scored the lowest, indicating nurses’ failure to attend to this domain (Diagram 1). The wards were merged into the general, office, and special department, and Tukey’s test was performed again, which revealed that the clinical reasoning score was the lowest in the special ward (26.94) and the highest (36.75) in the nursing office. The multivariate linear regression model was used in the multivariate analysis of factors relating to clinical reasoning based on the present study variables.

Table 3 presents the results of the regression analysis. The results showed that higher education level (B = 9.5, *P* = 0.018) and organizational position (B = 4.3, *P* = 0.017) were predicting factors for nurses’ clinical reasoning scores. Moreover, R2 = 0.086 for the final model meant that 8.6% of the changes in clinical reasoning score depend on academic qualification and organizational position, and based on analysis of variance, this model has significant goodness of fit (F = 7.185, df = 2,132, *P* = 0.001) (Table 3).

**Table 3:** Predicting nurses’ clinical reasoning score

Coefficients^a^									
Model		**Unstandardized Coefficients**		**Standardized Coefficients**	**Sig**	**95.0% Confidence Interval for B**		**Collinearity Statistics**	
		**B**	**Std. Error**	**Beta**		**Lower Bound**	**Upper Bound**	**Tolerance**	**VIF**
5	(Constant)	3.817	7.933		0.631	-11.879	19.512		
	Education (fog/lisanse)	9.496	3.968	0.203	0.018	1.645	17.346	0.961	1.040
	Post (super/nurse)	4.298	1.782	0.205	0.017	0.773	7.822	0.961	1.040

^a^Dependent Variable: TOTAL_SCORE, R^2^ = 0.086, Good ness of fit: F = 7.185, df =(2,132), *P* = 0.001

## Discussion

In this study, an attempt was made to evaluate nurses’ clinical reasoning based on the concepts of King’s theory. The mean score of clinical reasoning was below average for most nurses. Karimi-Naghd et al. investigated critical thinking and clinical decision-making in senior nursing students and working nurses and reported the mean clinical decision-making ability in nurses working in Sabzevar Hospital as 63.70 (ranging from 20 to 97), who scored lower compared to nursing students [24]. The results of a study by Shahraki titled “Clinical decision-making of ICU nurses in Mashhad teaching hospitals” showed that nurses had average clinical reasoning scores [25]. In a study by Lakdizgi et al. to investigate clinical decision-making of nurses working in teaching hospitals affiliated to Tabriz University of Medical Sciences, nurses had an average level of the clinical reasoning [26]. Nikoei investigated clinical decision-making in midwifery students, and reported their scores as average [27]. The review of 11 studies by Yogi showed that the results of 10 studies indicated the inadequacy of nurses’ clinical reasoning in the process of giving medication to patients [28]. A low clinical reasoning score in the present study may have been due to the small sample size. Also, 61.1% of the participants in this study had less than 10 years of work experience, which can have an impact on their clinical reasoning.

In the present study, nurses had a high mean clinical reasoning score in the domain of space, indicating its importance among nurses. Dehghani reported the importance of patient privacy from the perspective of the medical-care team of Shahr-e-Kord University of Medical Sciences, where 92% of the participants believed that respecting people’s privacy is an important concept and a basic human need [29]. Jahanpour, in a study investigating the perspective of patients and nurses on patient privacy while receiving nursing care reported an average level of observing human territory and personal space [30]. In the present study, the domain of authority and power based on King’s theory had the lowest clinical reasoning score. In this domain, the purpose of authority and power is the individual’s ability to use and mobilize resources to attain goals. The present study results show that nurses lack attention to this concept. Their “lack of attention” could be due to their lack of experience in providing complete care to hospitalized patients.

In a study investigating observing patient rights in Isfahan’s teaching hospitals, Akbari showed that patient rights were moderately observed [31]. In the present study, clinical reasoning score had a significant relationship with education level, organizational position, and service ward. Nurses with higher education levels had significantly higher clinical reasoning scores, which probably emphasizes teaching critical thinking at postgraduate levels. In their study, Montreu also reported that the clinical reasoning of nurses with master’s degrees was higher than that of nurses with bachelor’s degrees [32]. The results obtained by Shahraki agree with those of the present study regarding a significant relationship between clinical reasoning and organizational position, and that those in higher clinical positions had higher clinical reasoning scores [33]. Hoffman also reported a relationship between clinical position and clinical reasoning, which agrees with the present study results [34]. Considering that many factors are involved in selecting nurses as head nurses and supervisors such as their appropriate clinical reasoning and a high level of work experience as well as master’s degree qualification and management ability, all these factors appear to directly and indirectly affect their higher clinical reasoning scores. In the present study, clinical reasoning scores had no significant relationship with age, gender, or work experience. Scott et al. also reported no relationship between clinical reasoning and gender [35]. According to a study by Lee, nurses with longer work experience showed better clinical reasoning [36]. Based on the results of the present research, it seems necessary to use educational methods based on nursing models at all educational levels to promote clinical reasoning and critical thinking in educational and care systems. Also, creating a suitable organizational environment for providing clinical reasoning by working nurses, which requires the support of hospital officials, can improve the process of clinical reasoning. In this regard, continuous training programs for working nurses can also help strengthen this skill by placing them in different decision-making situations. In addition, due to the importance and usefulness of many theories, the gap between theory and practice has minimized their clinical application, while proper training and application of these theories can minimize this gap. Therefore, according to the placement of nurses in different decision-making situations and the need for clinical reasoning in those situations, scenarios following theories can help nurses in identifying the best solution in decision-making situations.

## Limitations and suggestions for future research

This study was conducted in a hospital in Iran, and its results cannot be generalized to nurses in all hospitals, it is suggested that this study be conducted in other medical training centers as well. Another limitation of this research is the lack of quantitative content validity of the research, which is due to the impossibility of using quantitative content validity for scenarios.

## Conclusion

Considering the importance of using theories as well as the importance of clinical reasoning in nursing decisions and their performance, it seems that teaching and using existing nursing theories can promote clinical reasoning and critical thinking of nurses. Therefore, educational planners should pay special attention to the use of these theories in the development of educational and clinical systems and use them as a clinical guide for healthcare services.

## Data Availability

All data generated or analyzed during this study are included in this published article.

## Declarations

All participants gave informed consent to participate in the study and voluntarily participated in the study.
